# Emotional challenges in neurodivergent children and young people: Progress and open questions for research and practice

**DOI:** 10.1002/jcv2.70109

**Published:** 2026-02-25

**Authors:** Giorgia Michelini, Alessio Bellato

**Affiliations:** ^1^ Centre for Brain and Behaviour, Department of Psychology, School of Biological and Behavioural Sciences Queen Mary University of London London UK; ^2^ School of Psychology University of Southampton Southampton UK; ^3^ Centre for Innovation in Mental Health University of Southampton Southampton UK; ^4^ Developmental Evidence Synthesis, Prediction, Implementation (EPI) Lab University of Southampton Southampton UK; ^5^ Institute for Life Sciences University of Southampton Southampton UK; ^6^ School of Psychology University of Nottingham Semenyih Malaysia; ^7^ South‐East Asia Mental Health Consortium (SEAMHC) University of Nottingham Semenyih Malaysia

**Keywords:** cognitive‐affective factors, emotional challenges, neurodivergence, social and contextual factors, transdiagnostic approaches

## Abstract

This Editorial introduces the March 2026 issue of *JCPP Advances*, marking the journal's sixth year with 15 impactful articles spanning key topics in child and adolescent mental health. A recurring theme across several articles is the investigation of emotional challenges—encompassing depression, anxiety, and self‐harm—particularly in the context of neurodivergence. These articles advance understanding of candidate cognitive‐affective drivers of emotional challenges, emphasise the role of social and contextual factors, and highlight the importance of research employing a transdiagnostic approach and informed by lived experience. Here, we underscore critical implications for assessment, prevention, and intervention, and reflect on outstanding challenges and future directions for research and clinical practice.

## Key Points

- Emotional challenges are common in neurodivergent children and young people, but their developmental pathways remain poorly understood.
- Several articles in this issue advance understanding of candidate cognitive‐affective and contextual factors underlying emotional challenges in this population, including intolerance of uncertainty, cognitive functioning, and emotional regulation vs. burden.
- Findings highlight the need for more inclusive, transdiagnostic approaches to identifying and supporting emotional challenges in neurodivergent children and young people.

This March 2026 issue of *JCPP Advances* marks the beginning of the journal's sixth year with a collection of innovative and rigorous articles spanning key topics in child and adolescent mental health, from attention deficit hyperactivity disorder (ADHD) to self‐harm to service use. One theme that stands out across multiple articles is the focus on emotional challenges—broadly defined, spanning depression, anxiety, and self‐harm—in the context of neurodivergence and related traits (Mareva et al., 2025; Nawaz et al., 2025; Ong et al., 2025; Sonuga‐Barke et al., 2025).

We use ‘neurodivergence’ here as an umbrella term encompassing autism, ADHD, and other neurodevelopmental conditions, in line with lived experience (Morris et al., 2025; Ostaszewska et al., 2025) and empirical evidence supporting neurodevelopmental clustering (Astle et al., 2022; Michelini et al., 2024). The focus on emotional challenges in neurodivergent children and young people directly addresses the research priorities of the neurodivergent community (Ostaszewska et al., 2025) and reflects the growing attention to the complex socio‐emotional and mental health needs of this population in clinical, school, and community settings.

As this topic has until recently been relatively under‐researched, we have a limited understanding of the factors that predict and drive the onset and maintenance of emotional challenges in neurodivergent children and young people (Caserini et al., 2025). This knowledge is crucial for developing effective treatment and preventative strategies. The study by Ong et al. (2025) exemplifies significant progress in this area. The authors report findings from a randomised controlled trial of a parent‐mediated group intervention designed to foster autistic young children's capacity to cope with uncertain situations that increase anxiety or distress. This intervention builds on research showing that intolerance of uncertainty is a common feature of autistic children with co‐occurring anxiety. The intervention group showed improvements in blinded assessors' ratings of children's responses to uncertainty after 8 weeks, with sustained improvements in this outcome, as well as in parent‐reported anxiety, at the subsequent 2‐month follow‐up. This study charts a clear roadmap for translational research in this area. Future work could focus on other cognitive‐affective factors that have been suggested to underlie emotional challenges in neurodivergent children and young people (Padaigaitė‐Gulbinienė et al., 2025).

In another paper in this issue, Mareva et al. (2025) used a person‐centred clustering approach in a sample of 1300 young people to delineate three clusters of mental health difficulties from mid‐ to late‐adolescence, and explored how cognition relates to developmental transitions in these mental health profiles. Difficulty adjusting risk‐related behaviour and poor spatial working memory in mid‐adolescence characterised youth with persistent ADHD and conduct symptoms (i.e., elevated at both mid‐ and late adolescent time points), but not those with resolving or emerging difficulties. Interestingly, youth with persistent ADHD and conduct symptoms also showed elevated emotional challenges in late adolescence, with no cluster showing ADHD/conduct symptoms alone at this later time point. As the identified cognitive factors appear to predict emerging emotional challenges in young people with ADHD/conduct symptoms, future research should test whether they represent promising targets for reducing this common co‐occurrence in youth.

Another candidate driver of emotional challenges in neurodivergent young people is emotional dysregulation. The registered report (RR) by Sonuga‐Barke et al. (2025)—*JCPP Advances*' very first RR—aims to test the mediating role of difficulties in emotional regulation in the longitudinal link between autistic/ADHD traits and depression measured 12 months later, and to contrast it with another candidate mediator: emotional burden. This new construct was informed by lived experience and defined as more frequent exposure to upsetting events that are experienced more intensely relative to neurotypical peers. While difficulties in emotional regulation emphasise regulatory deficits *within* the neurodivergent young person, the hypothesised role of emotional burden shifts our focus to the role of *external* adverse circumstances in neurodivergent young people's lives. This clear shift in perspective—closely aligned with the neurodiversity paradigm and previously promoted by our journal (Bellato & Seker, 2025)—can have profound implications for interventions to treat and prevent depression in neurodivergent young people. For example, rather than encouraging the neurodivergent young person to change their way of thinking and behaving (a common approach in cognitive‐behavioural therapy), focusing on emotional burden would promote interventions that seek to reduce exposure to upsetting events, for example, through school‐based approaches aimed at improving inclusion and acceptance of neurodivergent traits.

More generally, Sonuga‐Barke et al. (2025) highlight the importance of moving away from a primary focus on individual risk factors and towards greater attention to social and contextual factors. The study by Nawaz et al. (2025), based on data from more than 12,000 adolescents from the 2023 OxWell Student Survey, is another example of this important trend. The study investigated cross‐sectional associations between self‐harm and school experience measures, such as enjoyment and bullying, in addition to young people's demographic and clinical characteristics. Self‐harm was associated with students' perceptions that their school did not deal well with bullying and that they were unfairly picked on by teachers, among other factors. Of note, these experiences are significantly more common among neurodivergent children and young people (possibly contributing to emotional burden). As around half of the OxWell participants reporting self‐harm self‐identify as neurodivergent (Skripkauskaite et al., 2025), it is possible that these contextual correlates of self‐harm disproportionately affect neurodivergent young people, although future longitudinal research is needed to test this possibility.

Looking ahead, this issue's impactful work on emotional challenges in the context of neurodivergent traits gives us the opportunity to reflect on outstanding challenges in this area. One issue that we believe warrants greater attention is that neurodivergent children and young people may experience, describe, and manifest emotional challenges differently from neurotypical peers. Thus, their emotional challenges might not be adequately captured by formal diagnostic criteria and standardised assessments, which were largely developed based on how emotional challenges present in general population samples, often not including neurodivergent people. Neurodivergent traits, such as challenges with communication, abstract thinking, memory, and alexithymia (difficulty recognising emotions), can affect young people's experiences and reports of emotional challenges, as well as parent reports. It is also difficult to distinguish similar neurodivergent traits and emotional symptoms, such as social difficulties in autism and anxiety, particularly when relying on brief and decontextualised items in available questionnaires. Consequently, emotional challenges may be underestimated or misattributed to neurodivergent traits (i.e., diagnostic overshadowing). While some studies in autism and ADHD have begun to shine a light on neurodivergent presentations and lived experiences, more research is needed to inform assessment and clinical practice for emotional challenges in ways that are more inclusive of neurodivergent experiences.

Another outstanding issue is that we have a limited understanding of whether, and how, risk pathways to emotional challenges are similar or different in neurodivergent children and young people compared with neurotypical peers. Although the notion of equifinality (i.e., multiple possible developmental pathways leading to a given outcome) is generally accepted in our field, there has been relatively little research directly comparing neurodivergent and neurotypical groups, particularly using longitudinal designs and comprehensive assessments of risk factors. For example, going back to Sonuga‐Barke et al. (2025), the mediating role of emotional burden may point to a driver of depression that is largely specific to the lived experiences of neurodivergent young people.

Finally, while research is increasingly embracing transdiagnostic approaches, clinical and community services continue to focus predominantly on individual conditions, often reflecting the training and expertise of the professional conducting the assessment, with limited attention to co‐occurring difficulties. This undermines the identification of emerging emotional challenges in young people with a prior neurodevelopmental diagnosis, but can also lead to missed identification of ADHD or autism among young people assessed primarily for emotional challenges. Our hope is that increased use of transdiagnostic approaches in translational child and adolescent mental health research will promote more holistic clinical practices, recognising that co‐occurring conditions and intersecting needs are the rule rather than the exception in clinical and community settings. This perspective served as motivation for the 2026 Special Issue of *JCPP Advances* on transdiagnostic approaches that will be published later this year. Watch this space!

## AUTHOR CONTRIBUTIONS

**Giorgia Michelini**: Conceptualization; writing—original draft; writing—review and editing; **Alessio Bellato**: Writing—review and editing.

## CONFLICT OF INTEREST STATEMENT

G. M. and A. B. are joint editors of *JCPP Advances*.

## ETHICAL CONSIDERATIONS

Ethical approval was not required for this Editorial article.

## Data Availability

Data sharing not applicable to this article as no datasets were generated or analyzed in this Editorial article.
